# Stellate Ganglion Block for Intractable Hiccups Secondary to a Motor Vehicle Collision

**DOI:** 10.7759/cureus.37030

**Published:** 2023-04-02

**Authors:** Daniel J Lopez, Sanjeev Kumar

**Affiliations:** 1 Department of Pain Medicine, University of Florida College of Medicine, Gainesville, USA

**Keywords:** sympathetic ganglion, ultrasound-guided, stellate ganglion block, pain, hiccups

## Abstract

Intractable repetitive hiccups are a rare prolongation of the common physiologic reflex arc. If left untreated, chronic hiccups can decrease a patient's quality of life. Many nonpharmacologic, pharmacologic, and interventional treatment modalities have emerged. A 53-year-old male with a past medical history of a motor vehicle collision (MVC) two years earlier presented to a pain clinic with hiccups lasting several months. The patient was experiencing weight loss, lack of sleep, mood changes, and aspiration pneumonia requiring hospitalization secondary to the hiccups. Vagal and respiratory maneuvers and multiple prescription drugs failed to offer hiccup cessation. An ultrasound-guided stellate ganglion block offered immediate, prolonged cessation of the hiccups. When nonpharmacologic and pharmacologic therapies fail to offer improvement of hiccups, as in our patient's case, a stellate ganglion (SG) block may be a viable treatment option for medically refractory cases.

## Introduction

Intractable medically refractory hiccups are rare and defined as lasting longer than one month [1]. In cases of refractory hiccups, morbidity in the form of exhaustion, anorexia, dehydration, insomnia, and acquired mood disorders exists [2]. These prolonged symptoms can be debilitating and decrease the quality of life for patients suffering from this recurrent reflex. Many causes of persistent hiccups have been identified, including pathology of the central or peripheral nervous system, gastrointestinal system, respiratory tract, metabolic system, and psychosomatic system. Several treatment options may provide temporary or permanent cessation of recurrent hiccups, including vagal and respiratory maneuvers, pharmacologic therapy, and nerve blockade or stimulation [3]. Three case studies have introduced the stellate ganglion (SG) block as a potentially effective treatment for hiccups secondary to surgery, stroke, abdominal organ damage, and central nervous system pathology [4-6]. We present a case of ultrasound-guided SG block for the treatment of intractable hiccups. To our knowledge, this is the first case of an SG block resulting in the cessation of medically refractory and intractable hiccups secondary to a motor vehicle collision. Written informed consent was obtained from the patient.

## Case presentation

A 53-year-old man with a past medical history of a motor vehicle collision (MVC) presented to a pain clinic with persistent medically refractory hiccups two years after the MVC. The patient sought medical care from specialists including a neurologist, otolaryngologist, and gastroenterologist. Work-up, including cervical spine and brain MRIs, were negative for pathology, such as but not limited to neural injury. He was treated with chlorpromazine, gabapentin, and cyclobenzaprine, which caused undesired side effects such as drowsiness and dizziness. Vagal maneuvers such as the oculocardiac reflex, carotid sinus massage, and the Valsalva maneuver failed to resolve the hiccups. Despite pharmacotherapy and vagal maneuvers, the patient's hiccups occurred multiple times per minute for several months. The patient experienced a lack of sleep, difficulty breathing, vomiting, and weight loss secondary to the persistent hiccups. Additionally, he was hospitalized for aspiration pneumonia leading to hypoxia secondary to his refractory hiccups. Given the patient's morbidity directly associated with the refractory hiccups, he was offered an SG block.

**Figure 1:**
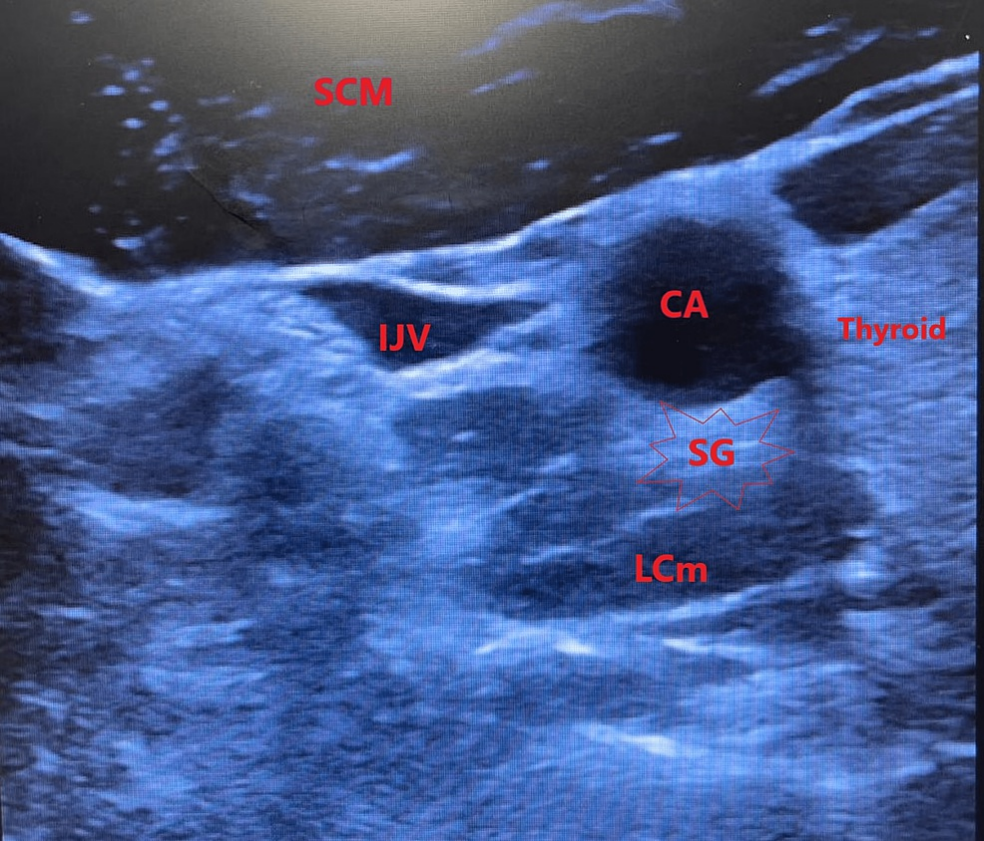
Sonographic image of the anatomy displaying the right IJV, right CA, right LCm, right SCM, right SG, and thyroid gland IJV - internal jugular vein, CA - carotid artery, LCm - longus coli muscle, SCM - sternocleidomastoid muscle, SG - stellate ganglion

A unilateral, right-sided, ultrasound-guided SG block was chosen due to the higher safety profile compared to a left-sided block. The risk of a pneumothorax is decreased given the lower apex of the lung compared with the left. Additionally, there is a decreased risk of hitting the thoracic duct and recurrent laryngeal nerve when compared with performing a left-sided SG block. The SG block was performed in an outpatient pain clinic using aseptic precautions. The patient was placed in a supine position. Using ultrasound guidance, the vertebrae of C6 and C7 were identified to locate the C6 uncinate process. Other nearby structures, such as the carotid artery, internal jugular vein, longus coli muscle, and thyroid gland, were also identified, displayed in Figure 1.

**Figure 2:**
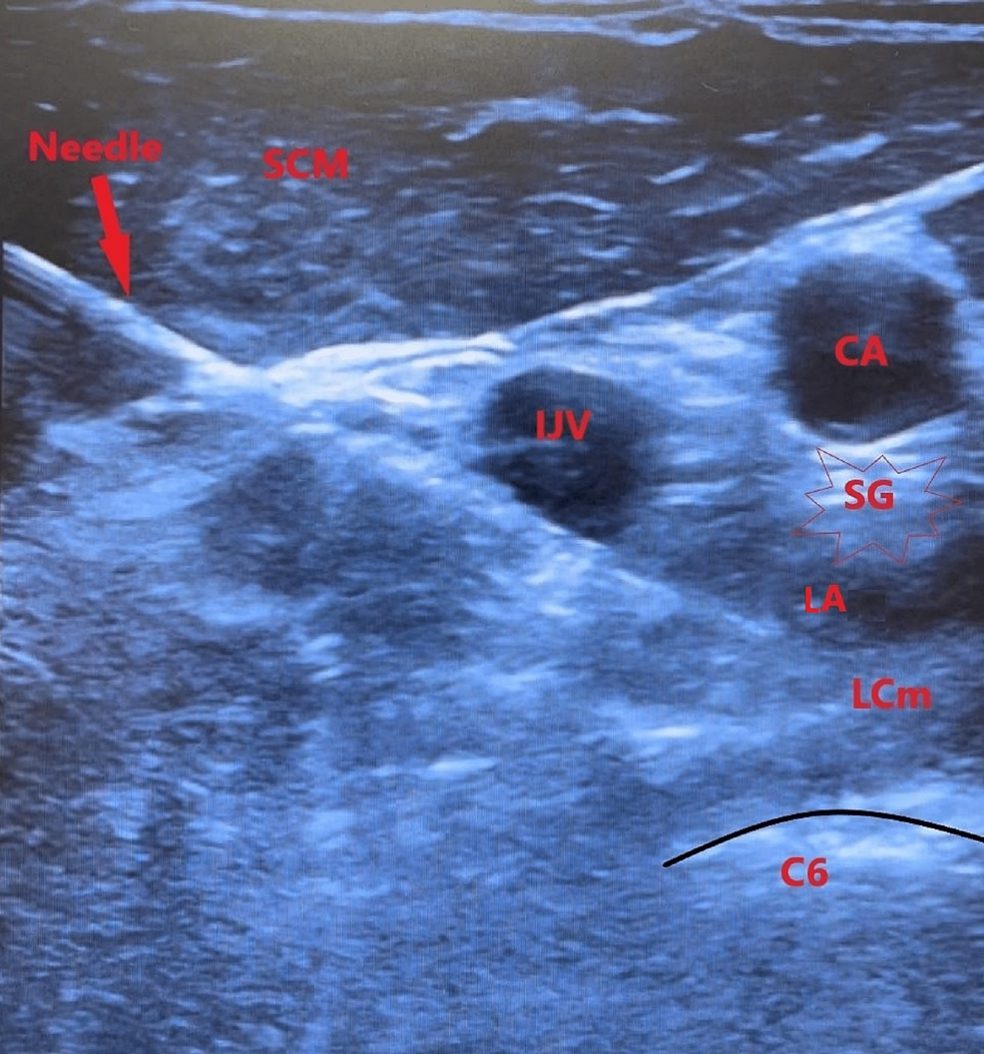
Sonographic image of the anatomy and in-plane ultrasound-guided stellate ganglion block displaying the needle with LA spread, sixth cervical vertebra (C6), right IJV, right CA, right LCm, right SCM, and right SG LA - local anesthesia, IJV - internal jugular vein, CA - carotid artery, LCm - longus coli muscle, SCM - sternocleidomastoid muscle, SG - stellate ganglion

Using a 5 cm, 25-gauge spinal needle, an in-plane approach was used to contact the bone slightly anterior to the C6 uncinate process. The spinal needle was retracted approximately 1 mm, and after negative aspiration, 4 mL of 4% lidocaine was incrementally injected at the level of the SG superficial to the longus coli muscle with visualization of the local anesthetic spread around the SG, displayed in Figure 2.

The decision to utilize 4% lidocaine was based off studies showing potential neurolytic effects when using higher concentrations as well as using less volume to achieve the nerve block [7]. The success of the SG block was also confirmed when the patient exhibited miosis of the right pupil and ptosis of the right upper eyelid (Horner syndrome), which resolved after several hours. The procedure provided immediate and complete cessation of the hiccups. At follow-up appointments, the patient reported an overall increased quality of life with improved sleep, resolved dyspnea, and regained appetite. Hiccup cessation lasted for approximately five months after the procedure. The patient returned for a repeat SG block, which also offered hiccup cessation for more than four months.

## Discussion

Hiccups are continual spontaneous contractions of the diaphragm and intercostal musculature that result in the inspiration of air and subsequent glottis closure, causing an abrupt blockade to inspiratory air and producing a "hiccup-like" sound [3]. Hiccups can be categorized into three distinct classes based on duration [4]: acute hiccups last less than 48 hours; persistent hiccups last more than 48 hours but less than one month; and intractable hiccups last more than one month.

The pathophysiology of hiccups is thought to occur as part of a reflex arc encompassing the central, afferent, and efferent neural pathways. Literature suggests that the afferent limb consists of the phrenic, vagus, and thoracic sympathetic nerves [3,8]. According to a review by Friedman, the efferent limb is mainly driven by the phrenic nerve [8]. The central neural pathways consist of the cervical spinal cord regions, brainstem reticular formation and respiratory centers, and hypothalamus [3]. The γ-aminobutyric acid (GABA)-ergic and dopaminergic neural pathway transmissions have also been linked to the pathophysiology of hiccups.

Hiccups may originate from a broad range of organ and nervous system pathology in addition to postsurgical, toxicologic, psychogenic, and pharmacologic etiologies. Additionally, any insult to the parasympathetic or sympathetic nerves can presumably precipitate this reflex arc, such as nerve irritation or injury [4]. Our patient suffered from intractable hiccups, which can be detrimental to a patient's quality of life secondary to interrupted sleep, fatigue, and difficulty with alimentation. These symptoms can cause both physical consequences, such as weight loss and dehydration, in addition to psychological ailments, such as depression [3].

In cases of intractable hiccups, treatment options range from nonpharmacologic therapy to interventional therapy. Nonpharmacologic options range from vagal stimulation to respiratory maneuvers. Pharmacologic approaches to hiccups focus on the proposed GABAergic and dopaminergic pathways. These include dopamine-altering drugs such as chlorpromazine (a first-line treatment approved by the US Food & Drug Administration), metoclopramide, and baclofen. GABA-altering drugs such as benzodiazepines and gabapentin have been effective in hiccup cessation [5]. In our patient's case, nonpharmacologic and pharmacologic treatment attempts failed to offer cessation of the hiccups. In this specific patient population with refractory intractable hiccups, interventional procedures targeting the vagus and phrenic nerves have been established as effective treatment options [2,9].

The SG, also known as the cervicothoracic ganglion, is a sympathetic ganglion formed by the fusion of the inferior cervical and superior thoracic ganglions [10]. It receives sympathetic input and transmits sympathetic output via the preganglionic and postganglionic fibers, respectively, for the head, neck, and superior limbs [10]. The SG is thought to play a key role in sympathetically mediated pain of the upper body, vasodilation, cardiovascular electrical changes, hidrosis physiology, and neurotransmitter regulation in the central nervous system [10-12]. The SG block has been shown to be effective in the treatment of complex regional pain syndrome, hot flashes, refractory ventricular arrhythmia, and posttraumatic stress disorder [11,12]. The role of the SG as part of the "reflex arc" of a hiccup is thought to be the reason why providing neural inhibition to the SG halts the hiccup.

To date, three published case reports have demonstrated the successful treatment of hiccups with an SG block. Lee et al. described three cases of postoperative hiccups lasting for three to six days that were treated successfully with an SG block [4]. Son et al. demonstrated cessation of persistent hiccups secondary to lateral medullary syndrome with an SG block [5]. Finally, Yamazaki et al. effectively performed an SG block or epidural block on seven patients with persistent hiccups secondary to central nervous system pathology, postoperative abdominal surgery, and idiopathic causes [6]. Our case of successful SG block was performed in a patient suffering from chronic intractable hiccups (i.e., lasting >1 month) secondary to a motor vehicle collision. To our knowledge, this is the first reported case of an SG block that offered cessation of medically refractory intractable hiccups caused by a motor vehicle collision.

Although rare, this relatively efficient and well-documented sympathetic block is not void of potential complications. These include vascular injury, pneumothorax, injury of the SG or other adjacent nerve structures via direct puncture, thyroid and tracheal damage, esophageal injury, systemic anesthetic toxicity via direct intravascular injection, and infections [10]. However, with careful preprocedural preparation, correct identification of anatomic landmarks via sonography, and knowledge of each potential complication, these negative consequences may be avoided. The risks and benefits of an SG block in a patient with hiccups should be carefully considered. The clinical, economic, and patient burden of acute and chronic hiccups is devastating and can adversely affect the quality of life for this population [13].

## Conclusions

Hiccups are a common physiologic phenomenon. Although they are mostly benign, in rare cases, they can pose grave complications for patients. When nonpharmacologic and pharmacologic therapies fail to offer improvement, as in our patient's case, an SG block may be an additional viable treatment option for medically refractory cases. This relatively simple and effective bedside procedure can offer cessation of hiccups, leading to a resolution of symptoms and increased quality of life. The physiology of the sympathetic nervous system and the role of the SG remains unknown. More research is needed to further explore the potential role of SG in the disease processes and the potential indications for SGBs in these patients.

## References

[1] Cymet TC (2002). Retrospective analysis of hiccups in patients at a community hospital from 1995-2000. J Natl Med Assoc.

[2] Tariq K, Das JM, Monaghan S, Miserocchi A, McEvoy A (2021). A case report of Vagus nerve stimulation for intractable hiccups. Int J Surg Case Rep.

[3] Steger M, Schneemann M, Fox M (2015). Systemic review: the pathogenesis and pharmacological treatment of hiccups. Aliment Pharmacol Ther.

[4] Lee AR, Cho YW, Lee JM, Shin YJ, Han IS, Lee HK (2018). Treatment of persistent postoperative hiccups with stellate ganglion block: three case reports. Medicine (Baltimore).

[5] Son H, Cho Y, Kim Y, Shin Y (2018). Stellate ganglion block for the treatment of intractable hiccups - a case report. Anesth Pain Med.

[6] Yamazaki Y, Mimura M, Iwasaki F, Hazama K, Namiki A (1998). Treatment of intractable hiccups with epidural block and stellate ganglion block. J Jpn Soc Pain Cl.

[7] Kalichman MW, Moorhouse DF, Powell HC, Myers RR (1993). Relative neural toxicity of local anesthetics. J Neuropathol Exp Neurol.

[8] Friedman NL (1996). Hiccups: a treatment review. Pharmacotherapy.

[9] Kang KN, Park IK, Suh JH, Leem JG, Shin JW (2010). Ultrasound-guided pulsed radiofrequency lesioning of the phrenic nerve in a patient with intractable hiccup. Korean J Pain.

[10] Piraccini E, Munakomi S, Chang KV (2022). Stellate Ganglion Blocks. https://www.statpearls.com/ArticleLibrary/viewarticle/29476.

[11] Tian Y, Wittwer ED, Kapa S (2019). Effective use of percutaneous stellate ganglion blockade in patients with electrical storm. Circ Arrhythm Electrophysiol.

[12] Lipov EG, Joshi JR, Sanders S, Slavin KV (2009). A unifying theory linking the prolonged efficacy of the stellate ganglion block for the treatment of chronic regional pain syndrome (CRPS), hot flashes, and posttraumatic stress disorder (PTSD). Med Hypotheses.

[13] Hendrix K, Wilson D, Kievman MJ, Jatoi A (2019). Perspectives on the medical, quality of life, and economic consequences of hiccups. Curr Oncol Rep.

